# Spin-communication channels between Ln(III) bis-phthalocyanines molecular nanomagnets and a magnetic substrate

**DOI:** 10.1038/srep21740

**Published:** 2016-02-24

**Authors:** A. Candini, D. Klar, S. Marocchi, V. Corradini, R. Biagi, V. De Renzi, U. del Pennino, F. Troiani, V. Bellini, S. Klyatskaya, M. Ruben, K. Kummer, N. B. Brookes, H. Huang, A. Soncini, H. Wende, M. Affronte

**Affiliations:** 1 Centro S3, Istituto Nanoscienze - CNR, via G. Campi 213/A, 41125 Modena, Italy; 2 Faculty of Physics and Center for Nanointegration Duisburg-Essen (CENIDE), University of Duisburg-Essen, Lotharstraße 1, D-47048 Duisburg, Germany; 3 Dipartimento di Scienze Fisiche, Informatiche e Matematiche, Università di Modena e Reggio Emilia via G. Campi 213/A, 41125 Modena, Italy; 4 Institute of Nanotechnology, Karlsruhe Institute of Technology (KIT), D-76344 Eggenstein-Leopoldshafen, Germany; 5 Institut de Physique et Chimie des Matériaux de Strasbourg, UMR 7504 UdS-CNRS, 67034 Strasbourg Cedex 2, France; 6 European Synchrotron Radiation Facility (ESRF), 71 Avenue des Martyrs, 38043 Grenoble, France; 7 School of Chemistry, The University of Melbourne, 3010 Victoria, Australia

## Abstract

Learning the art of exploiting the interplay between different units at the atomic scale is a fundamental step in the realization of functional nano-architectures and interfaces. In this context, understanding and controlling the magnetic coupling between molecular centers and their environment is still a challenging task. Here we present a combined experimental-theoretical work on the prototypical case of the bis(phthalocyaninato)-lanthanide(III) (LnPc_2_) molecular nanomagnets magnetically coupled to a Ni substrate. By means of X-ray magnetic circular dichroism we show how the coupling strength can be tuned by changing the Ln ion. The microscopic parameters of the system are determined by *ab-initio* calculations and then used in a spin Hamiltonian approach to interpret the experimental data. By this combined approach we identify the features of the spin communication channel: the spin path is first realized by the mediation of the external (5d) electrons of the Ln ion, keeping the characteristic features of the inner 4 f orbitals unaffected, then through the organic ligand, acting as a bridge to the external world.

The continuous trend towards ever smaller devices and components is leading to the requirement of fine control at the atomic scale over materials and interfaces. The natural solution can be found in molecular materials, which can be produced in large amounts and whose properties can be tailored by a bottom-up approach. Single molecule magnets^1^,^2^, featuring the spin degree of freedom^3^, have been demonstrated to possess specific magnetic properties and exhibit quantum phenomena such that they have been proposed to be employed in classical as well as quantum spintronics applications^4^,^5^. The enabling challenge here is the possibility to design specific magnetic features directly at the synthetic level and exploit them through a smart interaction with the environment, for instance a magnetic or conducting substrate. This requires a fine tailoring of the molecular characteristics, with the contemporary preservation of the core molecular properties and the existence of a sizable magnetic interaction with the external world through the organic ligands.

While the magnetization of simple paramagnetic molecules is pinned by a magnetic substrate and can only mimic its behavior^6^,^7^,^8^,^9^, an intriguing case is represented by the TbPc_2_ molecule. Here the characteristic properties of the isolated system, such as the huge anisotropy barrier resulting from the “double-decker” structure in which the Tb ion is sandwiched by two phthalocyanines^10^, are essentially preserved while a sizable magnetic interaction with the substrate has been reported^11^,^12^,^13^. This optimally balanced coupling is obtained by combining a lanthanide center, with well localized f-orbitals, with suitable organic ligands, hosting delocalized π-type electrons.

The microscopic understanding of the key interactions, linking the specific molecular features with its experimentally observed behavior, is a challenging task. This, however, may prompt the development of hybrid molecular devices, such as supramolecular spin valves made of TbPc_2_ on carbon nanotubes^14^ or graphene^15^ or molecular spin transistors^16^,^17^. Along this line, previous results include the study of the superexchange interaction in chains of CoPc molecules by spin-flip spectroscopy^18^ and the modelling of the interaction between a magnetic molecule and a non-magnetic metallic surface with an anisotropic Kondo impurity by means of numerical renormalization group calculations^19^. When magnetic substrates are considered, their interplay with the molecular layers is also at the heart of the spin-interface problem^3^ and it is mandatory for using molecular units as probes on surfaces with exotic properties^20^.

We report here a X-ray absorption spectroscopy (XAS) and magnetic circular dichroism (XMCD) investigation on LnPc_2_ (Ln = Tb, Dy, Er) molecules deposited on Ni (111) single crystal. Since XMCD is element-sensitive, we can follow the magnetization of the Ln ions and Ni simultaneously. We observe that the strength of the magnetic molecule-substrate interaction changes with the Ln ion, proving the dependence on the degree of hybridization between the localized magnetic 4f-orbitals of the Ln and the delocalized π-type electrons of the Pc ligands. Experimental results are rationalized with the support of a full theoretical modelling, where the microscopic details of the molecular units are firstly estimated via *ab initio* methods, and then inserted in spin Hamiltonian used to fit the experimental curves and derive the values for the exchange coupling. By doing this, we identify the microscopic details of the spin path linking the Ln ion with the Pc ligand and then to the substrate.

We find that there is not a direct overlap between the localized 4f states and the Pc unit, but rather the interaction occurs through the auxiliary 5d orbitals of the Ln ion, which become spin-polarized by the 4f–electrons and hybridize with the Pc ligands π-orbitals. This three-step spin-communication channel [(4f)_Ln_ ↔ (5d)_Ln_ ↔ (Pc) ↔ surface] is responsible for the experimentally observed coupling between the Ln ions and their environment, yet preserving unaffected the pristine magnetic properties of the molecules.

## Results

### System characterization and high fields XMCD

**Figure 1:**
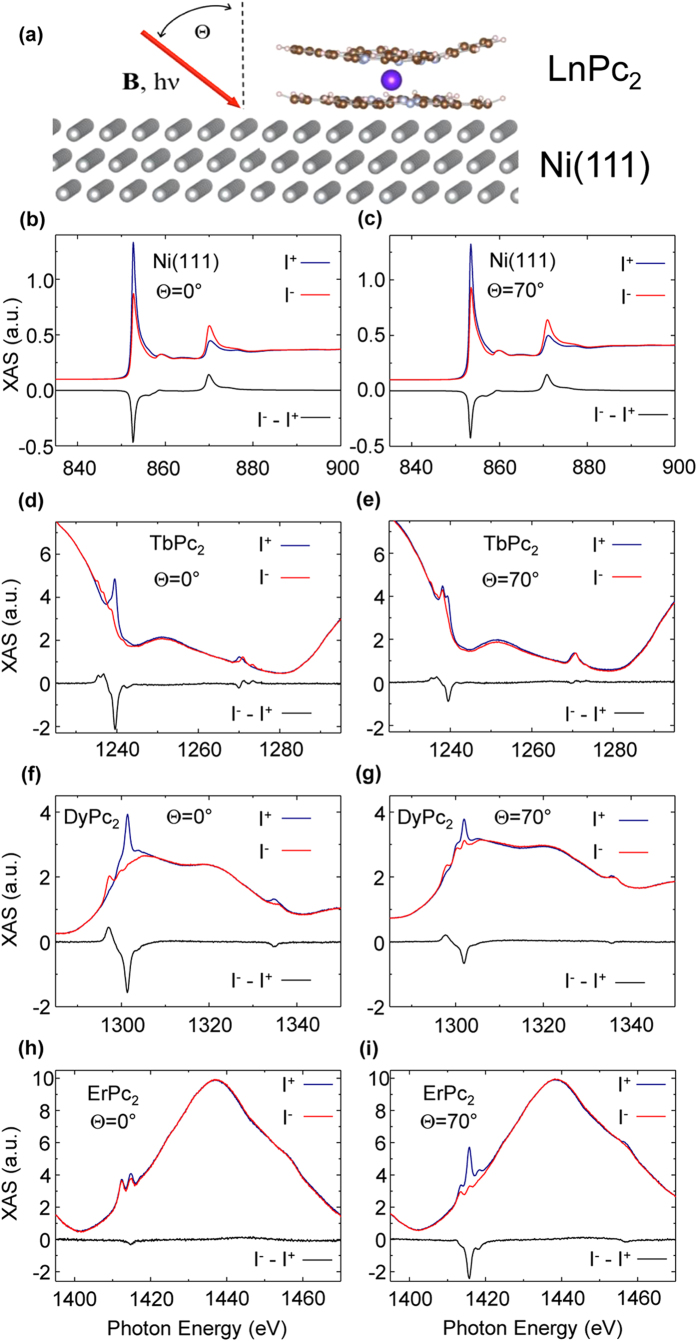
XAS and XMCD characterization of the system. (**a**) Schematic view of the experimental geometry; (**b–i),** XAS and XMCD spectra for Ni-*L*_2,3_ (**b,c**), Tb-*M*_4,5_ (**d,e**), Dy-*M*_4,5_ (**f,g**), Er-*M*_4,5_ (**h,i**) edges in LnPc_2_/Ni(111) at Θ  = 0° (left panels) and Θ = 70° (right panels) incidence angles, taken at an applied external field ***B*** = 5 T and temperature T = 8 K. XAS spectra have been normalized with respect to the average *L*_3_ (*M*_5_) intensity (I^+^ + I^−^)/2, which has been set to 1.

Figure 1(a) displays a schematic view of our experimental geometry. More details on the sample preparation and preliminary characterizations are given in the Method Section and Supplementary Notes 1–2 and Supplementary Figures 1–4. Briefly, the samples were prepared *in situ* by evaporating ~0.3 monolayers of LnPc_2_ molecules on the freshly prepared Ni(111) surface. STM analysis combined with XLD measurements (see Supplementary Figure 1 and Figure 3) confirm that the molecules adsorb in a flat geometry, with the Pc plane parallel to the surface^11^,^12^,^21^,^22^,^23^,^24^,^25^,^26^,^27^. XMCD measurements at the *L*_2,3_ absorption edges of Ni and the *M*_4,5_ absorption edges of Ln were performed in total electron yield mode at the ID08 beamline of the ESRF, Grenoble. The experimental configuration is such that the magnetic field ***B*** is always parallel to the incident photon beam, with an angle Θ with respect to the normal of the sample surface which can be rotated (see Fig. 1(a)). The base temperature during all of our measurements was 8 K.

Figure 1(b–i) shows the XAS and XMCD spectra of Ni-*L*_2,3_ edges and Ln-*M*_4,5_ edges for the LnPc_2_ molecules taken at normal and grazing incidence at a magnetic field ***B*** = 5 T. These spectra compare well with the ones already reported^11^,^12^,^21^,^22^,^23^,^24^,^25^,^26^,^27^, demonstrating the robust character of these molecules. The XMCD intensity taken at the Ln *M*_4,5_ edges strongly depends on the field direction for TbPc_2_ (Fig. 1(d–e)) and DyPc_2_ (Fig. 1(f–g)) as previously reported^11^,^12^,^21^,^22^,^23^,^24^,^25^,^26^,^27^, and also for ErPc_2_ (Fig. 1(h–i)): Tb and Dy show the maximum for Θ  = 0° while for Er the signal is vanishingly small and becomes larger for increasing Θ. These observations confirm that Tb and Dy have an easy axis of magnetization perpendicular to the molecular plane (Θ  = 0°) whilst Er exhibits an easy plane as observed in bulk Ln(III) bis-phathalocyanines^10^.

### Magnetization measurements

**Figure 2:**
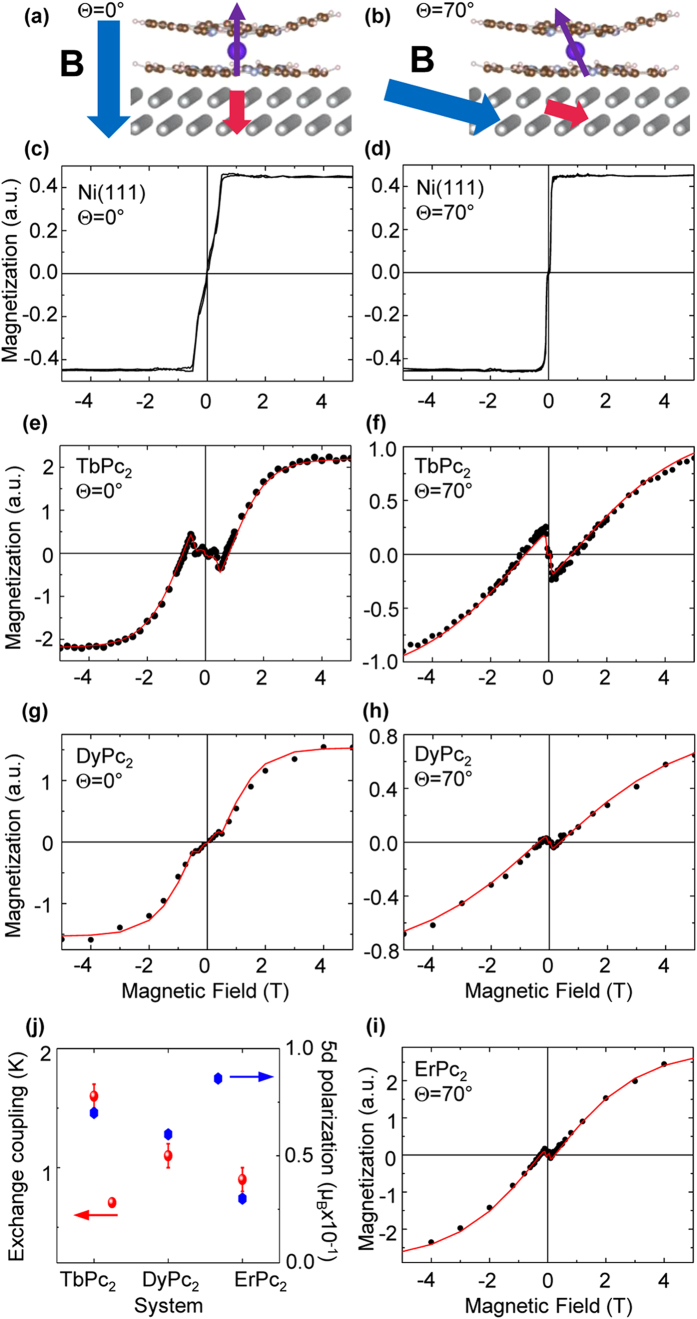
Magnetization cycles. **(a,b)** schematic representation of the magnetic coupling between the TbPc_2_ molecules and the Ni substrate in the low field region where the antiferromagnetic interaction prevails. At Θ  = 70° the magnetic moment possesses a (small) component parallel to the surface (usually negligible considering a perfectly axial V_cf_), as a result of the introduction of the low symmetry crystal field. **(c-i)** element-resolved magnetization measurements of Ni (**c,d**), Tb (**e,f**), Dy (**g,h**), Er (**i**) for the LnPc_2_/Ni(111) systems, taken at Θ  = 0° (left panels) and Θ  = 70° (right panels) incidence angles. Experimental data are shown as black dots, while the theoretical fit (as described in the text) as the continuous red line. (**j)** Exchange coupling constant *K* (left axis), as determined from the fit, and magnitude of the magnetic polarization (right axis) induced on the 5d orbitals for each Ln compound calculated from DFT as described in the text. The error bars in *K* take into account also the uncertainty in the experimental data.

In order to study the magnetic coupling between the molecules and the substrate, we performed isothermal magnetization cycles, which can be obtained by plotting the XMCD signals (see Supplementary Note 3 for more details) for each element as a function of the external magnetic field ***B*** at a fixed temperature (8 K in our measurements). In Fig. 2(e–i) we show the element-resolved magnetization-loop for each lanthanide, with the field applied perpendicular (left panels) and at grazing angle (right panels) to the sample surface. For the ErPc_2_ molecules, only the curve at Θ = 70° is shown, given the vanishingly small signal observed at normal incidence. Along with the Ln magnetization of the LnPc_2_ molecules, the corresponding magnetization for the Ni substrate is plotted in Fig. 2(c–d).

For high magnetic fields, both the molecules and the Ni moments are aligned parallel to the external field and follow a conventional magnetization curve. However, for small fields, the magnetization curves measured on the lanthanides are generally opposed to ***B***; in addition, they present an abrupt change of the slope in correspondence of the field *B*_s_ at which the Ni magnetization saturates (*B*_s_ = 0.5 T for Θ = 0° and 0.2 T for Θ = 70°). These features reveal a coupling of the lanthanide’s magnetic moment to the Ni substrate of antiferromagnetic character. Close to ***B*** = 0, the coupling with the underlying Ni crystal forces the lanthanide moment to be antiparallel to the substrate (see Fig. 2(a,b)), while at high fields the Zeeman interaction prevails and the magnetizations are all parallel to the external field. When these two interactions compensate each other, the magnetization of the molecule is zero, and the actual magnetic field value at which this condition is satisfied (which we call *B*_exc_) can be considered as a qualitative estimation of the coupling strength. We note that the magnetic coupling can be tuned by changing the Ln ion as the interaction strength displays a clear trend in going from TbPc_2_ to ErPc_2_. This can be better visualized considering for instance the values of *B*_exc_ at Θ = 70° for the three compounds, namely *B*_exc_ = 0.90 T, 0.35 T and 0.25 T for Tb, Dy and Er, respectively. We shall address this observation in the following sections.

### Theoretical modeling of the magnetic interaction

In order to proceed with a quantitative analysis of the Ln magnetization curves reported in Fig. 2 to extract information about the strength and character of the molecule-surface coupling, we modeled the system with the following spin Hamiltonian^11^:


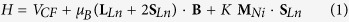


where **L**_*Ln*_ and **S**_*Ln*_ are the orbital and spin angular momentum operators of the 4f electrons, the second term describes the Zeeman interaction between the Ln total magnetic moment and the magnetic field, and the last term describes the exchange-like magnetic coupling between the lanthanide 4f-electronic spin **S**_*Ln*_ and the Ni magnetization **M**_*Ni*_ via the coupling constant *K*. The Ni magnetization **M**_*Ni*_ is normalized by taking the ratio with its saturation value, and it is taken from the experimental curves presented in Fig. 2(a–b)). Strong spin-orbit coupling and strong on-site electron repulsion within the 4f-shell are taken into account by projecting the Hamiltonian onto the Ln ground atomic spin-orbit multiplet 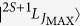, where *J*_MAX_ = *L* + *S* (i.e. |^7^*F*_6_ > for Tb^III^, |^6^*H*_15/2_ > for Dy^III^, and |^4^*I*_15/2_ > for Er^III^). Within such subspace, it is always true that **S**/S = **L**/L = **J**/J, i.e. the three operators are all equivalent, up to a multiplicative constant. Finally, *V*_CF_ is the microscopic crystal field potential exerted by the charge distribution of the ligands on the 4f electrons. Given the pseudo *D*_4*d*_ symmetry of LnPc_2_ molecules, it is customary to approximate *V*_CF_ to an axially symmetric electrostatic potential containing only three fitting parameters, which were first optimized by Ishikawa *et al.*^10^. However, such an approximation (as the X-ray structure has no formal local point-group symmetry, although not too far from an idealized *D*_4*d*_ structure) can be inaccurate especially when the angular dependence of the magnetization is considered^28^. Therefore, we perform a full *ab initio* calculation of all 27 crystal field parameters for the 4f electrons of the LnPc_2_ molecules, using the experimental (X-ray) structure measured for the TbPc_2_ compound in bulk^29^. The *ab initio* methodology to describe the crystal field spectrum in rare earth complexes is well established^30^,^31^,^32^ and it consists of the Complete Active Space Self-Consistent Field (CASSCF) and State-Interacting (RASSI) methodologies^32^, accounting for strong electron correlation and strong spin-orbit coupling, respectively (more details about the calculations are in the Methods section and in the Supplementary Note 4).

Inserting the optimized *ab initio* low-symmetry crystal field potential *V*_CF_ in the Hamiltonian of Equation (1), we obtain the fit reported as solid lines in Fig. 2, panels (e–i). The quality of the fit is remarkably good, despite the use of just a single fit parameter (the molecule-surface coupling constant *K*) plus a multiplicative factor which scales the computed value for the Ln magnetization with the measured XMCD intensity. The best fits are obtained for *K*_Tb_ ~ 1.6 K, *K*_Dy_ ~ 1.1 K and *K*_Er_ ~ 0.9 K, finding a quantitative estimation for the strength of the molecule-surface interaction and confirming the experimental observation that it depends on the specific Ln ion and decreases by going from TbPc_2_ to ErPc_2_. The visibility of this effect at the moderate temperature of 8 K should not be surprising, as the effect of the temperature is only to broad the thermal population of the eigenstates of Equation (1). At small external fields already sufficient to saturate the Ni substrate magnetization, the two lowest lying levels (which would be almost degenerate in the absence of coupling) will be separated by an energy roughly proportional to *S*_Ln_*K*_Ln_. As a consequence, the magnetization will deviate from a conventional curve up to temperatures even much higher than *K*.

Our fit procedure of the TbPc2 magnetization curve on Ni employs realistic crystal field parameters obtained from ab initio calculations, and without the introduction of a strongly anisotropic coupling interaction, which was recently proposed in Reference 11 to account for the experimental observation that *B*_exc_ at Θ = 70° for TbPc_2_ is (slightly) greater than *B*_exc_ at Θ = 0°. While the effect of low symmetry harmonics in the crystal field potential is indeed to mix the *M* = ±6 ground doublet with all the excited states (see Supplementary Note 4 for a description of the ground state wave functions) and also introduce J-mixing, only MJ-mixing was considered in the current approach, and this did not introduce significant improvements in the fit, although we expect that overall state mixing can be even more pronounced for the actual situation with molecules on substrate that are probably more distorted than what we estimated here.

It is possible that also other mechanisms contribute to the observed angular dependence of *B*_exc_. For instance, the role of the spin *s* = 1/2 π-vacancy delocalized over the Pc ligands of the molecules could be taken into account since the spin radical may be involved in linking the magnetic interaction between the Ln center and the magnetic substrate^33^,^34^.

### DFT calculations of the intramolecular Ln-Pc interaction

In our experiments we tune the LnPc_2_ – Ni interaction by changing the Ln ion inside the molecule. Since the organic ligand is the same for the three molecules, the character of the Ln orbital wave-function should play a key role in determining the magnetic anisotropy and interactions. We are therefore addressing the intramolecular Ln-Pc interaction only, corroborating the intuitive picture that the Ln-Ni coupling is indeed mediated by the organic part of the molecule.

To get a deeper insight on the intramolecular coupling, we have performed DFT calculations on TbPc_2_, DyPc_2_, ErPc_2_ molecules in gas phase with the Perdew-Burke-Ernzerhof (PBE)^35^ exchange correlation functional as implemented in Quantum Espresso^36^ including static correlation effects using the LDA + U formalism^37^. Further computational details are given in the Methods section.

**Figure 3:**
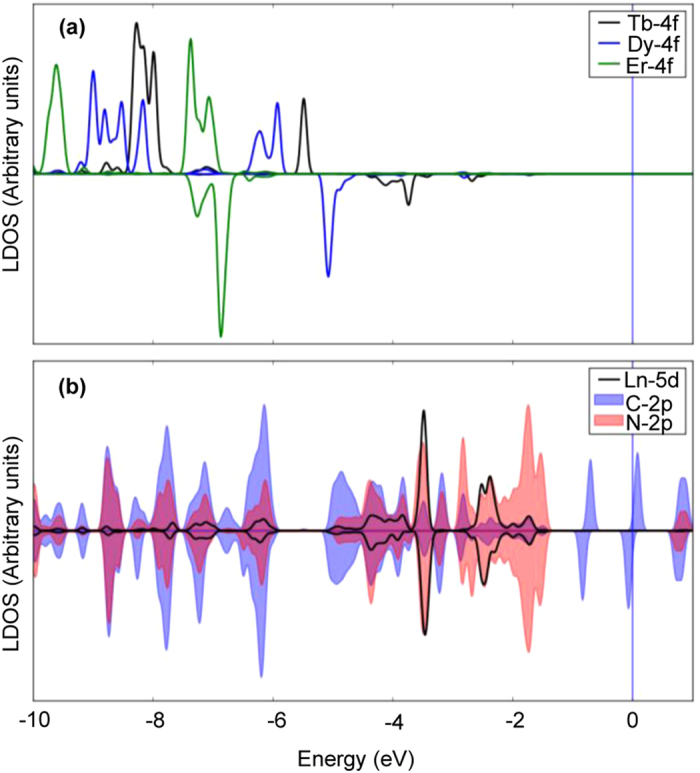
DFT calculation of spin-resolved local density of states (LDOS) of the LnPc_2_ molecules. (**a**) spin-resolved LDOS projected on the 4f orbitals of Ln = Tb, Dy and Er. (**b**) spin-resolved LDOS projected on the 5d orbital of Tb (the one for Dy and Er is very similar) and on the 2p orbitals of the nearest-neighbours (to the Ln ions) N and C atoms (which is only weakly modified by the choice of the Ln) in the phthalocyanine planes. In each panel, the upper (lower) side is relative to the majority (minority) spin channel. d-character in (**b**) has been enhanced for better comparison.

The calculated projected local density of states (LDOS) of the LnPc_2_ complexes are shown in Fig. 3, where we plot the states with *f* and *d* orbital character of the Ln ions, as well as the p-states of the N and C ions closer to the Ln ion, where is left most of the spin-polarization of the *s* = 1/2 spin radical delocalized over the two phthalocyanine parts.

Already for Tb, the *f* orbitals are well below the Fermi level, due to the inclusion of U, and, moving from to Dy to Er, progressive filling of the minority *f* states pushes the electronic levels further down in energy. We observe a partial occupation of the 5d orbitals together with a small spin polarization in all the three cases with the same sign of the *f* spin moments. This implies that there is a hybridization between the *f* and *d* states in the Ln ions, which in our LDOS becomes evident for Tb, in the region of −4 eV from E_F_ in the minority spin channel. The values of this induced polarization are plotted in Fig. 2(j), being 0.07 μ_B_, 0.06 μ_B_ and 0.03 μ_B_, respectively for the Tb, Dy and Er ions. We note that the trend going from Tb to Er is the same as observed experimentally for the coupling strengths. Since the Ln 5d orbitals occupy the same energy region of (and hybridize with) the p-states of the C and N atoms in the phthalocyanines, we conclude that such d-mediated interaction is the mechanism through which the spin information in the *f* orbitals propagates to the delocalized states of the two phthalocyanines, which, in turn, communicate to the external world by direct contact (in this case with the spin-polarized surface states). Our picture is in agreement with the one set forward in dinuclear complexes, where the role of 5d orbitals has been advocated in order to explain the exchange coupling through Oxygen between Gd and Cu ions^38^. We stress that this result has implications also for different studies focusing on LnPc_2_ and analogue compounds, where the magnetic coupling between the Ln ions to the external world is relevant^13^,^14^,^15^,^16^,^17^ and, more generally, could help in the realization of novel Ln-based molecular magnets^39^.

## Conclusions

We systematically studied how the interaction between LnPc_2_ single ion magnets and a ferromagnetic substrate is tuned by changing the Ln ion. With the support of composite *ab initio*, DFT, and spin Hamiltonian theoretical and computational approaches, we have identified the microscopic origin of the spin communication channels between the central Ln ion and the magnetic substrate, and characterized it as a three-step process: the f-electrons hybridize with the 5d orbitals of the metal ion, which in turn act as a bridge to the delocalized states in the organic part of the molecules, ultimately mediating the communication with the magnetic surface. We employed an *ab initio* calculated low-symmetry crystal field potential in our spin Hamiltonian to reproduce the experimental results evaluating the strength *K* of the magnetic coupling between lanthanide ion and surface, which is found to decrease in going from Tb to Dy to Er. This is explained in terms of the modulation of the hybridization between (4f) ad (5d) orbitals on these metal ions, as reproduced by the DFT calculations. These systems constitute a test bed for more general models which aim at a microscopic and quantitative description of the magnetic interactions between individual molecular compounds and magnetic substrates.

## Methods

### Sample preparation and synchrotron measurements

Experiments were carried out at the ID08 beamline of the European Synchrotron Radiation Facility (ESRF) in Grenoble, France. The Ni(111) single crystal (Surface Preparation Laboratory) was used as the substrate. Before molecule deposition, the surface was cleaned by repeated cycles of Ar^+^ sputtering (Energy = 2 KeV for 20 minutes and E = 0.8 KeV for 10 minutes) and annealing (Temperature = 800 °C for 5 minutes). The quality of the surface was checked by Low Energy Electron Diffraction (LEED). A ~0.3 monolayer of LnPc_2_ molecules was evaporated after long degassing of the powders, keeping the evaporator temperature at 420 °C at a base pressure of 1.0 × 10^−9^ mbar and monitoring the thickness with an *in situ* quartz microbalance. XMCD measurements at the *L*_2,3_ absorption edges of Ni and the *M*_4,5_ absorption edges of Ln were performed in total electron yield mode. The magnetic field ***B*** was applied parallel to the incident photon beam, at an angle Θ with respect to the normal of the sample surface (see Fig. 1(a) for a schematic picture).

The STM investigation as well as further characterization of our sample (linear dichroism absorption measurements, *ex situ* XPS and Raman spectroscopies) are presented in Supplementary Notes 1–2 and Supplementary Figures 1–4.

### DFT calculations

We performed our calculations using the projector augmented wave method (PAW)^40^ as recently implemented in the pseudopotentials made by Andrea Dal Corso^41^. We have taken into account the influence of the on-site Coulomb interaction imposing static correlation effects on the f electrons of the Ln ions^36^ (values of U and J have been taken by ref. 42, we used the same value of U = 9 eV and J = 1 eV for the Tb, Dy and Er ions).

No spin-orbit coupling is included in the calculation. We modeled our systems with a tetragonal supercell of 20 × 20 X 14 Å. Regarding the kinetic and charge density cutoffs, we have chosen 121 and 620 Ry, 125 and 499 Ry, 135 and 578 Ry, respectively for TbPc_2_, DyPc_2_ and ErPc_2_. A smearing parameter of 8 × 10^−4^ Ry ensured a correct description of the spin radical in the two Pcs. The version of Quantum Espresso used in all the calculations was the 5.0.3.

### Ab-initio strategy for the evaluation of the crystal field parameters of the LnPc_2_ molecules

We perform an explicitly correlated full *ab initio* calculation of the crystal field states in TbPc_2_ using the software MOLCAS 8.0^32^. As we did not impose any symmetry in the crystal field, we employed the experimental (X-ray) structure measured for the TbPc_2_ compound in bulk^29^. It is important to remark that the main finding of our approach (i.e. the activation of the new tunneling mechanisms) is a consequence of the introduction of the low symmetry harmonics and it is therefore very robust with respect to the microscopic details of the molecular structures used in the calculations. First we separately optimize state-averaged Complete Active Space Self-Consistent Field (SA-CASSCF) wavefunctions for all relevant spin symmetries obtained by the Ln-ion f electrons in the active space composed of the seven 4 f atomic orbitals. The optimization takes into account scalar-relativistic Douglas-Kroll Hamiltonian as implemented in the software MOLCAS 8.0. The resulting multiconfigurational wavefunctions, having a well-defined spin symmetry, are then non-perturbatively mixed by suitable spin-orbit Hamiltonian accounting for spin-other-orbit interactions in a mean-field manner (AMFI).

The split J-multiplet thus obtained is then projected onto a full crystal field Hamiltonian, with Stevens operators quantized along the principal magnetic axis obtained from direct calculation of the ground state g-tensor, which provides all 27 crystal-field parameters evaluated *ab initio*^31^. Such approach is of course sensitive to the choice of basis set, but previous experience has shown that using the relativistically-corrected ANO-RCC-DZP basis set on the lanthanide ion, and an ANO-RCC-DZ basis set on the lighter elements, leads to quite accurate results, especially when the ground doublet is well-known from experiments to be very close to a pure atomic state^30^. This is well known to be the case for the three molecules studied here. More details on the ab-initio calculation of the crystal field parameters are given in Supplementary Note 4.

## Additional Information

**How to cite this article**: Candini, A. *et al.* Spin-communication channels between Ln(III) bis-phthalocyanines molecular nanomagnets and a magnetic substrate. *Sci. Rep.* **6**, 21740; doi: 10.1038/srep21740 (2016).

## Supplementary Material

Supplementary Information

## References

[b1] Gatteschi D., Sessoli R. & Villain J. Molecular Nanomagnets (Oxford University Press, Oxford, 2006).

[b2] Kahn O. Molecular Magnetism (Wiley – VCH, New York, 1993).

[b3] Sanvito S. Molecular spintronics: the rise of spinterface science. Nat. Phys. 6, 562 (2010).

[b4] Bogani L. & Wernsdorfer W. Molecular spintronics using single-molecule magnets. Nat. Mater. 7, 179–186 (2008). 18297126 10.1038/nmat2133

[b5] Leuenberger M. N. & Loss D. Quantum computing in molecular magnets. Nature 410, 789–793 (2001). 11298441 10.1038/35071024

[b6] Wende H. *et al.* Substrate-induced magnetic ordering and switching of iron porphyrin molecules. Nat. Mater. 6, 516–520 (2007). 17558431 10.1038/nmat1932

[b7] Bernien M. *et al.* Tailoring the nature of magnetic coupling of Fe-porphyrin molecules to ferromagnetic substrates. Phys. Rev. Lett. 102, 047202 (2009). 19257470 10.1103/PhysRevLett.102.047202

[b8] Javaid S. *et al.* Impact on interface spin polarization of molecular bonding to metallic surfaces. Phys. Rev. Lett. 105, 077201 (2010). 20868071 10.1103/PhysRevLett.105.077201

[b9] Klar D. *et al.* Oxygen-tuned magnetic coupling of Fe-phthalocyanine molecules to ferromagnetic Co films. Phys. Rev. B 88, 224424 (2013).

[b10] Ishikawa N., Sugita M., Okubo T., Tanaka N., Iino T. & Kaizu Y. Determination of Ligand-Field Parameters and f-Electronic Structures of Double-Decker Bis(phthalocyaninato)lanthanide Complexes. Inorg. Chem. 42, 2440–2446 (2003). 12665381 10.1021/ic026295u

[b11] Lodi Rizzini A. *et al.* Coupling Single Molecule Magnets to Ferromagnetic Substrates. Phys. Rev. Lett. 107, 177205 (2011). 22107576 10.1103/PhysRevLett.107.177205

[b12] Lodi Rizzini A. *et al.* Exchange Biasing Single Molecule Magnets: Coupling of TbPc2 to Antiferromagnetic Layers. Nano Lett. 12, 5703–5707 (2012). 23046484 10.1021/nl302918d

[b13] Schwöbel J. *et al.* Real-space observation of spin-split molecular orbitals of adsorbed single-molecule magnets. Nat. Commun. 3, 953 (2012). 22805560 10.1038/ncomms1953

[b14] Urdampilleta M., Klyatskaya S., Cleuziou J.-P., Ruben M. & Wernsdorfer W. Supramolecular spin valves. Nat. Mater. 10, 502–506 (2011). 21685902 10.1038/nmat3050

[b15] Candini A., Klyatskaya S., Ruben M., Wernsdorfer W. & Affronte M. Graphene Spintronic Devices with Molecular Nanomagnets. Nano Lett. 11, 2634–2639 (2011). 21648452 10.1021/nl2006142

[b16] Vincent R., Klyatskaya S., Ruben M., Wernsdorfer W. & Balestro F. Electronic read-out of a single nuclear spin using a molecular spin transistor. Nature 488, 357–360 (2012). 22895342 10.1038/nature11341

[b17] Thiele S., Balestro F., Ballou R., Klyatskaya S., Ruben M. & Wernsdorfer W. Electrically driven nuclear spin resonance in single-molecule magnets. Science 344, 1135–1138 (2014). 24904159 10.1126/science.1249802

[b18] Chen X. *et al.* Probing Superexchange Interaction in Molecular Magnets by Spin-Flip Spectroscopy and Microscopy. Phys. Rev. Lett. 101, 197208 (2008). 19113306 10.1103/PhysRevLett.101.197208

[b19] Höck M. & Schnack J. Numerical Renormalization Group calculations of the magnetization of isotropic and anisotropic Kondo impurities. Phys. Rev. B 87, 184408 (2013).

[b20] Brede J. *et al.* Long-range magnetic coupling between nanoscale organic–metal hybrids mediated by a nanoskyrmion lattice. Nat. Nanotech. 9, 1018–1023 (2014). 10.1038/nnano.2014.235 25326693

[b21] Stepanow S. *et al.* Spin and Orbital Magnetic Moment Anisotropies of Monodispersed Bis (Phthalocyaninato) Terbium on a Copper Surface. J. Am. Chem. Soc. 132, 11900–11901 (2010). 20698538 10.1021/ja105124r

[b22] Margheriti L. *et al.* X-Ray Detected Magnetic Hysteresis of Thermally Evaporated Terbium Double-Decker Oriented Films. Adv Mater. 22, 5488–5493 (2010). 20949539 10.1002/adma.201003275

[b23] Biagi R. *et al.* X-ray absorption and magnetic circular dichroism investigation of bis(phthalocyaninato) terbium single-molecule magnets deposited on graphite. Phys. Rev.B 82, 224406 (2010).

[b24] Gonidec M. *et al.* Surface Supramolecular Organization of a Terbium(III) Double-Decker Complex on Graphite and its Single Molecule Magnet Behavior. J. Am. Chem. Soc. 133, 6603–6612 (2011). 21486019 10.1021/ja109296c

[b25] Vitali L. *et al.* Electronic Structure of Surface-supported Bis(phthalocyaninato) terbium(III) Single Molecular Magnets. Nano Lett. 8, 3364–3368 (2008). 18800852 10.1021/nl801869b

[b26] Klar D. *et al.* Hysteretic behaviour in a vacuum deposited submonolayer of single ion magnets. Dalton Trans. 43, 10686–10689 (2014). 24875369 10.1039/c4dt01005a

[b27] Klar D. *et al.* Antiferromagnetic coupling of TbPc_2_ molecules to ultrathin Ni and Co films. Beilstein J. Nanotechnol. 4, 320–324 (2013). 23766956 10.3762/bjnano.4.36 PMC3678430

[b28] Luzon J. & Sessoli R. Lanthanides in molecular magnetism: so fascinating, so challenging. Dalton Trans. 41, 13556–13567 (2012). 22936346 10.1039/c2dt31388j

[b29] Katoh K. *et al.* Direct Observation of Lanthanide(III)-Phthalocyanine Molecules on Au(111) by Using Scanning Tunneling Microscopy and Scanning Tunneling Spectroscopy and Thin-Film Field-Effect Transistor Properties of Tb(III)- and Dy(III)-Phthalocyanine Molecules. J. Am. Chem. Soc. 131, 9967–9976 (2009). 19569681 10.1021/ja902349t

[b30] Chibotaru L. F., Ungur L. & Soncini A. The Origin of Nonmagnetic Kramers Doublets in the Ground State of Dysprosium Triangles: Evidence for a Toroidal Magnetic Moment. Angew. Chem. Int. Ed. 47, 4126–4129 (2008). 10.1002/anie.200800283 18428177

[b31] Chibotaru L. F. & Ungur L. Ab initio calculation of anisotropic magnetic properties of complexes. I. Unique definition of pseudospin Hamiltonians and their derivation. J. Chem. Phys. 137, 064112 (2012). 22897260 10.1063/1.4739763

[b32] Veryazov V., Widmark P., Serrano-Andrés L., Lindh R. & Roos B. O. MOLCAS as a development platform for quantum chemistry software. International Journal of Quantum Chemistry 100, 626–635 (2004).

[b33] Komeda T. *et al.* Observation and electric current control of a local spin in a single-molecule magnet. Nat. Commun. 2, 217 (2011). 21364556 10.1038/ncomms1210 PMC3072104

[b34] Urdampilleta M., Klyatskaya S., Ruben M. & Wernsdorfer W. Magnetic Interaction Between a Radical Spin and a Single-Molecule Magnet in a Molecular Spin-Valve. ACS Nano 9, 4458–4464 (2015). 25858088 10.1021/acsnano.5b01056

[b35] Perdew J. P., Burke K. & Ernzerhof M. Generalized Gradient Approximation Made Simple. Phys. Rev. Lett. 77, 3865 (1996). 10062328 10.1103/PhysRevLett.77.3865

[b36] Giannozzi P. *et al.* QUANTUM ESPRESSO: a modular and open-source software project for quantum simulations of materials. J. Phys.: Condens. Matter 21, 395502 (2009). 21832390 10.1088/0953-8984/21/39/395502

[b37] Liechtenstein A., Anisimov V. I. & Zaanen J. Density-functional theory and strong interactions: Orbital ordering in Mott-Hubbard insulators. Phys. Rev. B 52, R5467 (1994). 10.1103/physrevb.52.r5467 9981806

[b38] Rajaraman G., Totti F., Bencini A., Caneschi A., Sessoli R. & Gatteschi D. Density functional studies on the exchange interaction of a dinuclear Gd(iii)-Cu(ii) complex: method assessment, magnetic coupling mechanism and magneto-structural correlations. Dalton Trans. 17, 3153–3161 (2009). 19421617 10.1039/b817540c

[b39] Sorace L., Benelli C. & Gatteschi D. Lanthanides in molecular magnetism: old tools in a new field. Chem Soc Rev. 40, 3092–3104 (2011). 21390351 10.1039/c0cs00185f

[b40] Blochl P. E. Projector augmented-wave method. Phys. Rev. B 50, 17953 (1994). 10.1103/physrevb.50.17953 9976227

[b41] www.qe-forge.org/gf/project/pslibrary.

[b42] Larson P., Lambrecht W. R. L., Chantis A. & van Schilfgaarde M. Electronic structure of rare-earth nitrides using the LSDA + U approach: Importance of allowing 4f orbitals to break the cubic crystal symmetry. Phys. Rev. B 75, 045114 (2007).

