# Supplementary material for: Spin-communication channels between Ln(III) bis-phthalocyanines molecular nanomagnets and a magnetic substrate
Source: Sci Rep. 2016 Feb 24;6:21740. doi: 10.1038/srep21740 (PMC4764849; doi:10.1038/srep21740)
Supplement: Supplementary Information [file srep21740-s1.pdf]

Supplementary Information for

Spin-communication channels between Ln(III) bis-phthalocyanines molecular nanomagnets and a magnetic substrate

Andrea Candini[1], David Klar[2], Simone Marocchi[1], Valdis Corradini[1], Roberto Biagi[1,3], Valentina de Renzi[1,3], Umberto del Pennino[1,3], Filippo Troiani[1], Valerio Bellini[1], Svetlana Klyatskaya[4], Mario Ruben[4,5], Kurt Kummer[6], Nicholas B. Brookes[6], Haibei Huang[7], Alessandro Soncini[7], Heiko Wende[2], Marco Affronte[1,3]

[1]Centro S3, Istituto Nanoscienze - CNR, via G. Campi 213/A , 41125 Modena. Italy.

[2]Faculty of Physics and Center for Nanointegration Duisburg-Essen (CENIDE), University of Duisburg-Essen, Lotharstraße 1, D-47048 Duisburg, Germany

[3]Dipartimento di Scienze Fisiche, Matematiche e Informatiche, Università di Modena e Reggio Emilia via G. Campi 213/A , 41125/A Modena. Italy.

[4]Institute of Nanotechnology, Karlsruhe Institute of Technology (KIT), D-76344 Eggenstein-Leopoldshafen, Germany

[5]Institut de Physique et Chimie des Mat´eriaux de Strasbourg, UMR 7504 UdS-CNRS, 67034 Strasbourg Cedex 2, France

[6]European Synchrotron Radiation Facility (ESRF), Avenue des Martyrs 71, 38043 Grenoble, France

[7]School of Chemistry, The University of Melbourne, 3010 Victoria, Australia

Supplementary Note 1: Experimental details on $LnPc_2$ films preparation and characterization

Experiments were carried out at the ID08 beamline of the European Synchrotron Radiation Facility in Grenoble, France. The Ni(111) single crystal was used as the substrate. Before molecule deposition, the surface was cleaned by repeated cycles of Ar^+ sputtering (Energy = 2 keV for 20 minutes and E = 0.8 keV for 10 minutes) and annealing (Temperature = 800 °C for 5minutes). The quality of the surface was checked by Low Energy Electron Diffraction (LEED). A ~ 0.3 monolayer of $LnPc_2$ molecules was evaporated after long degassing of the powders, keeping the evaporator temperature at 420 °C at a base pressure of 1.0 x 10^{-9} mbar and monitoring the thickness with an *in situ* quartz microbalance.

After the deposition of the molecules on the substrate, STM images show isolated spots with reproducible lateral size of 2-3 nm and height of 0.3-0.4 nm, compatible with the molecule sizes, assuming that the Pc ring lay flat on the surface (see Supplementary Figure 1(a-d), where the case of $TbPc_2$ is shown). From a statistical analysis applied to the STM images we derived that about 20–40% of the surface is occupied by a 2D distribution of isolated clusters.

By means of XPS, we have also investigated the chemical composition of the $LnPc_2$ molecules deposited on the Ni(111) surface. In Supplementary Figure 2 the core levels of the $TbPc_2$ /Ni(111) interfaces for two different coverage of the $TbPc_2$ deposited by sublimation are shown. Core level intensities have been analyzed taking into account the atomic sensitivity and the attenuation of the electronic signals. The Tb-3d, N-1s and C-1s core level line shapes measured for all the depositions fit well with the corresponding data obtained on a thick film deposited from the liquid phase (not shown). The N-1s/Tb-3d = 18±5 and C-1s/Tb-3d = 75±20 ratios are well reproducible and close to the expected ones (16 and 64), clearly indicating that the overall molecular stoichiometry is preserved during the heating and deposition processes. From the Tb-3d/Ni-2p ratio and by taking into account the Ni signal attenuation due to the overlayer, we obtained the average area occupied by one $TbPc_2$. Assuming that the complete coverage is made by molecules lying flat on the surface and considering an area of 2 nm^2 for each molecule, we derived a thickness of 0.3-0.5 ML for the $TbPc_2$ film, in agreement with the quantity read by the quartz microbalance and with the coverage derived by STM (20–40%).

XMCD measurements at the $L_{2,3}$ absorption edges of Ni and the $M_{4,5}$ absorption edges of Ln were performed in total electron yield mode. The magnetic field $\boldsymbol{B}$ was applied parallel to the incident photon beam, at an angle Θ with respect to the normal of the sample surface (see Figure 1(a) of the main paper for a schematic picture). Supplementary Figure 3 shows X-ray Linear Dichroism (XLD) on the N K and Ln $M_{4,5}$ edges which are found in agreement with what reported in previous works where $TbPc_2$[1,2,3] and metal-Pc[4,5] were deposited on substrates, indicating that the $LnPc_2$ molecules are isolated and flat on the substrate, with the Pc plane parallel to the surface.

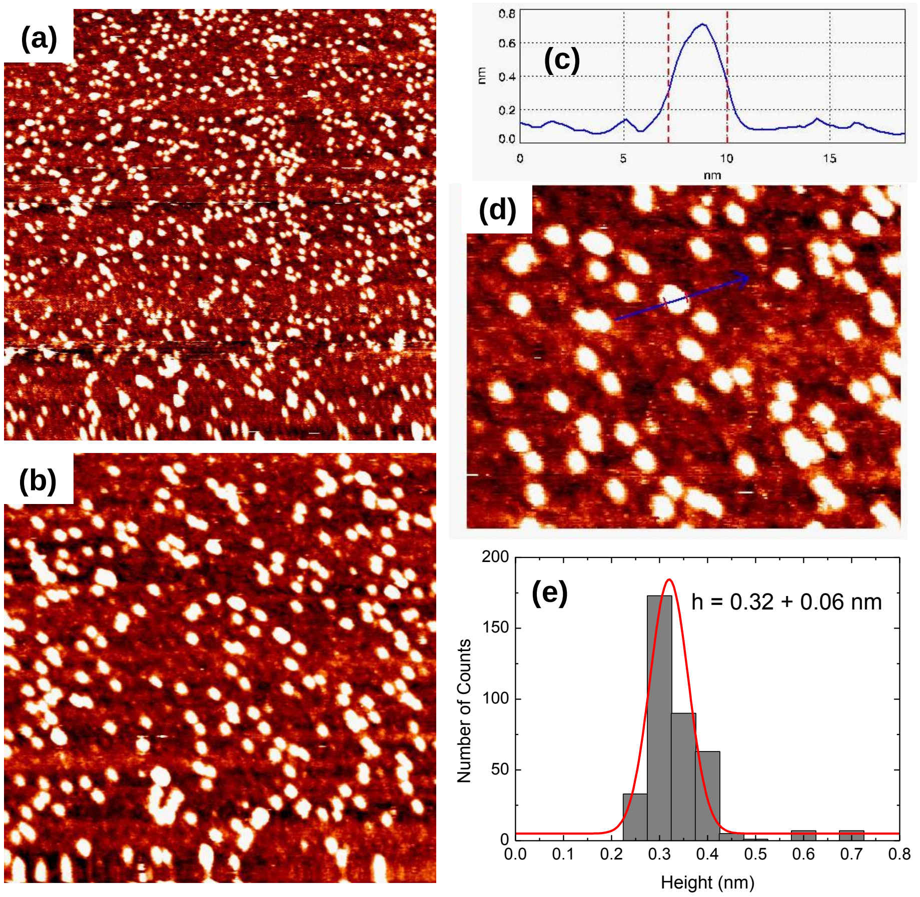

Supplementary Figure 1: STM characterization of the molecule film.

(a) 200x200nm^2, (b) 100x100nm^2 STM images of the $TbPc_2$ molecules on Ni(111). (c) Typical height profile measured along the line in panel (d) 30x20nm^2. (e) Histogram plot of the height profiles of more than 300 molecules.

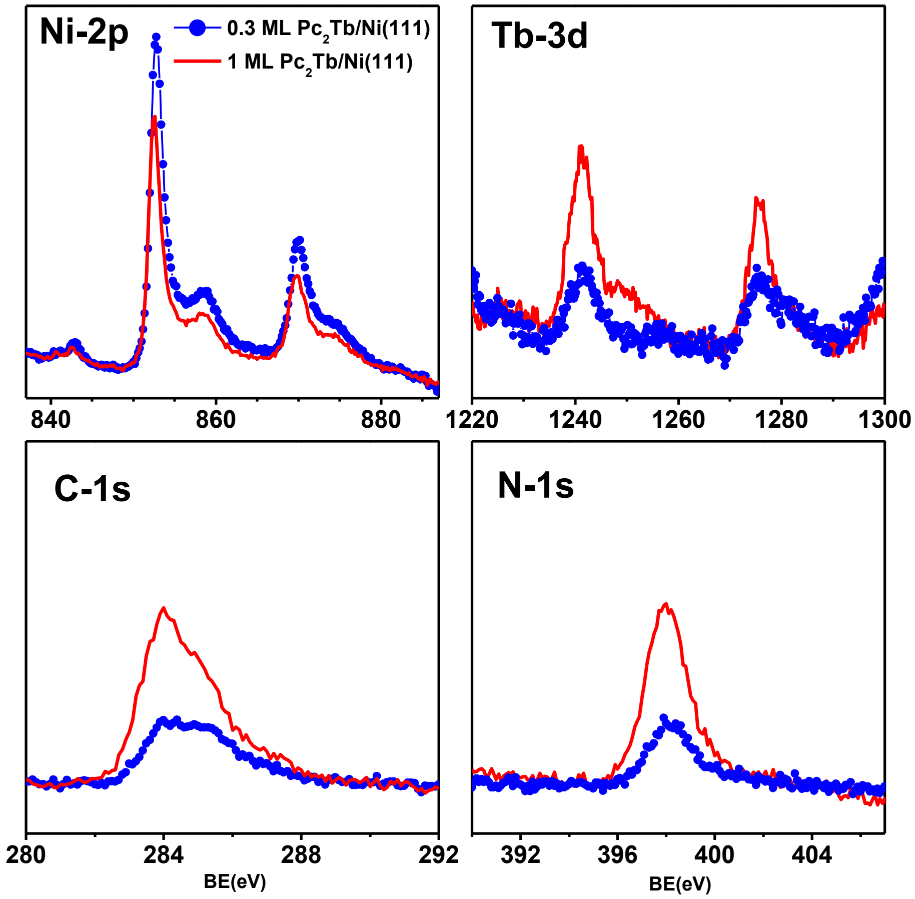

Supplementary Figure 2: XPS characterization of the molecular film.
XPS core levels for the $TbPc_2$ deposited by sublimation on the Ni(111) surface for two different coverages (0.3ML and 1ML).

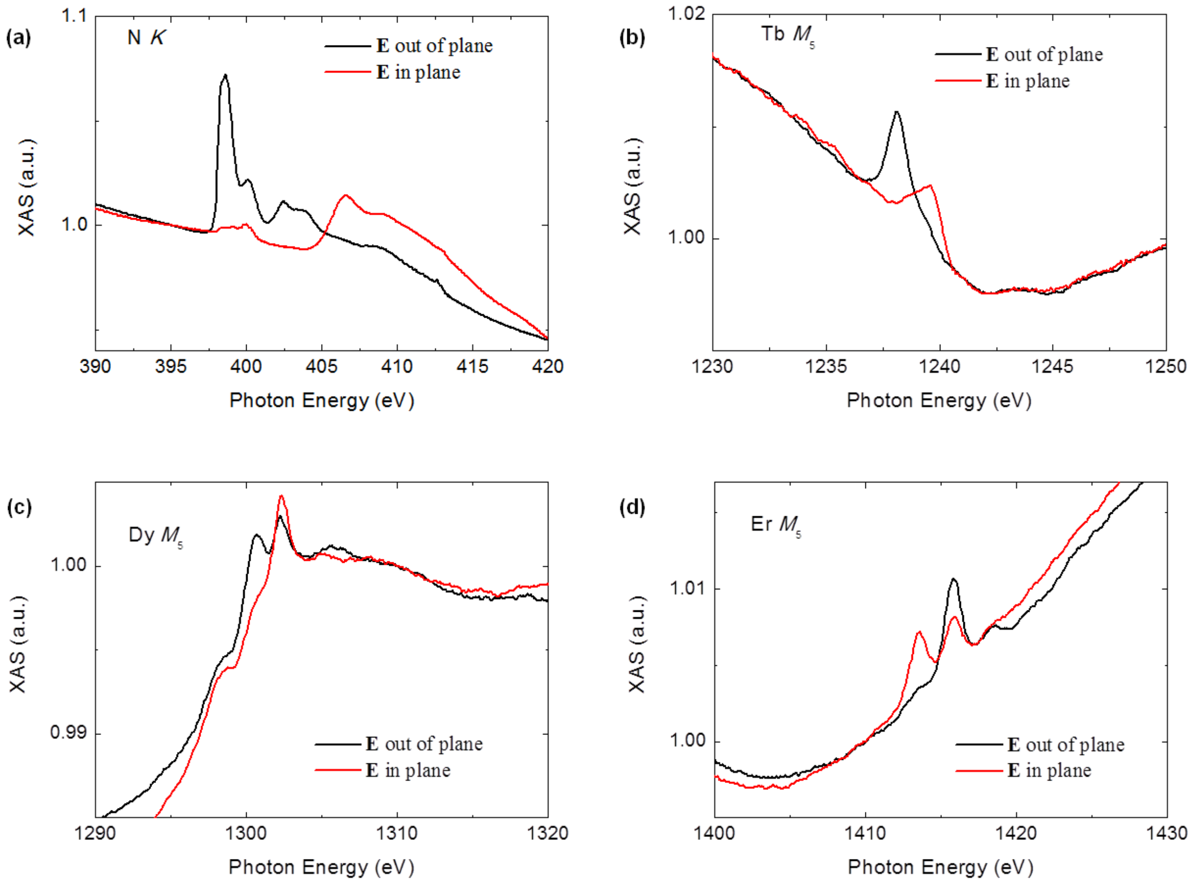

Supplementary Figure 3: X-ray linear dichroism for the LnPc$_2$ on Ni(111) systems.

X-ray linear dichroism at the (a) N *K* edge and (b-d) Ln M_5 edge (Ln = Tb, Dy, Er).

Supplementary Note 3: Checking the integrity of the TbPc$_2$ molecules deposited on Ni by Raman spectroscopy.

It was previously reported that TbPc$_2$ double decker may decompose into two phthalocyanine halves when deposited on Au(111) metal surface[6,7]. Similar results were found in Reference 1 on Cu(100), where pure intact TbPc$_2$ films were obtained with a careful degassing of the powders. To check the deposition on Ni(111) substrate, we performed extensive STM analysis on the film of TbPc$_2$ (the same conclusion is still valid for the other Ln derivatives), shown in Supplementary Figure 1(e). Although our STM set up has not enough resolution to clearly distinguish eight or four lobes corresponding to TbPc$_2$ and TbPc respectively, we reproducibly found the height of the molecule to be of 0.3 nm, without any signature of the presence of two different molecules species.

In addition, we also performed Raman spectroscopy on the submonolayer molecule film evaporated on Ni(111), shown in Supplementary Figure 4. Raman spectra clearly show the presence of all the peaks associated with the thin film TbPc$_2$ molecules at 1140, 1302, 1335, 1425, 1450 and 1515 cm^{-1}

as previously reported in Reference 8 for molecules deposited on graphene from solution, while the peak associated with the Pc species[9] at 1540 cm^{-1} is absent.

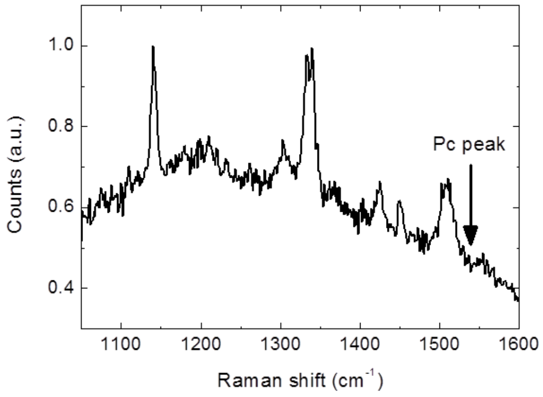

Supplementary Figure 4: Raman spectroscopy of $TbPc_2$ on Ni.

Raman spectrum of the $TbPc_2$ molecules evaporated as described in the main text on Ni(111). We did not observe the peak associated with the presence of isolated Pc moieties.

Supplementary Note 3: Details on XMCD measurements.

XMCD measurements at the at the $L_{2,3}$ absorption edges of Ni and $M_{4,5}$ absorption edges of Ln (Ln = Tb, Dy, Er) were performed in total electron yield mode. The base pressure in the measurement chamber was 1.0 x 10^{-10} mbar. An external magnetic field $\boldsymbol{B}$ can be applied parallel to the incident photon beam with an angle Θ with the sample surface (Θ = 0 defines the normal incidence direction). The dichroic spectrum is the difference between the XAS spectra taken with the helicity of the incident photon antiparallel (I^-) and parallel (I^+) to the external field. In order to minimize the effects of field inhomogeneity, we carried out measurements by switching both the helicity and the applied field. The final XMCD values are obtained by normalizing the difference $I^- - I^+$ by the height of the XAS edge. To plot the magnetization curve as a function of the external field, we recorded the XMCD intensity at the different fields. In the case of Ni and Tb, since the XAS and XMCD line-shapes do not change with the field, in order to make faster data acquisition for each field point we measured only the intensities of the L_3(M_5) edge (E) at 853(1243) eV and pre-edge (P) at 845(1232) eV for the two polarizations for each element under investigation; the resulting magnetization value is defined as: $(E^-/P^- - E^+/P^+)$ / ½ $(E^-/P^- + E^+/P^+)$. Due to technical issues concerning the stability vs time of the monochromator, this procedure was not possible for Dy and Er, where complete XMCD spectra have been taken for each field point.

Supplementary Note 4: Ab initio determination of the energies, wavefunctions and crystal field parameters for $LnPc_2^-$ molecules.

We performed explicitly correlated CASSCF/RASSI/SINGLE_ANISO calculations of ground and excited states in $TbPc_2$, $DyPc_2$ and $ErPc_2$ according to the methodology described in[10-13] as implemented in the software MOLCAS 8.0[14]. In the calculation we used the experimental structure published for $TbPc_2$ for all three molecules[6,15]. It is important to remark that the main finding of our approach (i.e. the activation of the new tunneling mechanisms) is a consequence of the introduction of the low symmetry harmonics and it is therefore very robust with respect to the microscopic details of the molecular structures used in the calculations. The split J-multiplet obtained from such calculations can then be projected onto a full crystal field Hamiltonian for the f-orbital space, which will contain 27 parameters evaluated ab initio[12]. Although the approach is in principle sensitive to the choice of the atomic Gaussian basis set and the number of CASSCF spin states non-perturbatively mixed in the RASSI module by an atomic mean-field integral (AMFI) spin orbit Hamiltonian[16,17], previous experience has shown that using the ANO-RCC-DZP basis set on the lanthanide ion, and ANO-RCC-DZ on the lighter elements, where the ANO-RCC basis sets are optimized for the description of scalar relativistic effects within the Douglas-Kroll theory as implemented in MOLCAS 8.0[14], leads to quite accurate results. To keep the approach simple, we only explored the optimization and spin-orbit mixing of all CASSCF states whose spin symmetry corresponds to the highest allowed spin state consistent with the 4*f*-orbital occupation. Thus for $TbPc_2$ we considered all the $S = 3$ CASSCF states, for $DyPc_2$ all the $S=5/2$ CASSCF states, and for $ErPc_2$ all the $S=3/2$ CASSCF states.

We summarize the resulting energy spectra, wavefunction-projection on the relevant multiplet basis, and resulting ab initio crystal field parameters, in four Supplementary Tables (ST's), where ST1 reports the 27 crystal field parameters optimized for the three molecules, and ST2, ST3 and ST4 report the energies and wavefunction decomposition of all the crystal field states for $TbPc_2$, $DyPc_2$ and $ErPc_2$, respectively. For the cases of $DyPc_2$ and $ErPc_2$ our results are in good agreement (within up to 3% of discrepancies) with previous calculations[15], despite we used slightly different approximations. Projection onto the |JM> multiplet basis assumes that the total angular momentum J is quantized along the principal magnetic axis, obtained from the diagonalization of the ab initio g-tensor for the relevant ground doublet.

Ground state of TbPc$_2$: As evident from table ST2, although the ground doublet is strongly dominated by axial components, we now have clear tunneling between $\pm M$ components and contributions from all other M-states. Note that the ground state is strongly dominated by the $M = \pm 6$ angular momentum component with a large gap to first excited state (although a bit smaller than Ishikawa's gap), consistent with the SMM properties of this molecule.

Ground state of DyPc$_2$: As evident from table ST3, the ground Kramers doublet is dominated by |±13/2> (88%) consistent with Ishikawa's pure axial picture, with contributions from |±15/2> (9%) and |±11/2> (3%) due to the more realistic low-symmetry treatment of the crystal field. The first excited state is at 78cm^{-1} from the ground state (to be compared with the Ishikawa excited state at 33cm^{-1}), and is dominated by |±11/2> (87%) as predicted by the Ishikawa's model.

Ground state of ErPc$_2$: The ground Kramers doublet is dominated by |±1/2> (99.8%) consistent with Ishikawa's pure axial picture, with contributions from |±9/2> (0.2%) due to the more realistic low-symmetry treatment of the crystal field. The first excited state is at 59cm^{-1} from the ground state (to be compared with the Ishikawa excited state at 102cm^{-1}), and is dominated by |±3/2> (96.8%) as predicted by the Ishikawa's model.

k	q	Tb	Dy	Er
2	-2	0.45677950479964E+00	-0.37078608123151E+00	-0.67520112754540E-04
	-1	-0.18999653120307E+00	0.79188689822427E+00	-0.12646943274601E-03
	0	-0.70588871561054E+01	-0.32682271889236E+01	0.13936206054344E+01
	1	-0.17577594581546E+00	0.15297855439202E+01	0.66041795063412E-04
	2	0.25423979757607E+00	0.21845452226366E+00	-0.18298159748576E-03
4	-4	-0.13856367886294E-02	-0.12436653160727E-02	0.54744822288353E-02
	-3	0.57026472457533E-02	0.62640713388655E-03	-0.14475516600373E-04
	-2	-0.94742339423831E-03	0.19411512659610E-02	-0.33903004387382E-06
	-1	0.21151228682653E-02	-0.96814426257610E-02	0.47541903103704E-05
	0	-0.12354687542473E-01	0.82984893946984E-02	-0.56725639072316E-02
	1	0.24073400334739E-02	-0.17965578076213E-01	-0.14346680612705E-04
	2	-0.22883315162448E-02	0.52680548767083E-03	0.21127186607624E-05
	3	-0.14823354343031E-02	-0.12315242838304E-02	0.10112289759647E-04
	4	0.16872079185591E-01	0.26321656430736E-02	-0.13374639411667E-02

6	-6	0.14615964840185E-04	-0.16699826098596E-05	-0.74052591339402E-07
	-5	-0.49919041988232E-04	0.31973662711979E-04	0.24365163685034E-05
	-4	0.42238043334871E-05	0.12291842855368E-04	0.21456094219083E-03
	-3	0.18830657298135E-04	0.49304270279584E-05	-0.23794596393478E-06
	-2	-0.12772909783601E-04	0.66709729111433E-05	0.32289489930409E-07
	-1	-0.50637778430637E-05	-0.61201110834332E-04	-0.17385957788489E-07
	0	0.36378138924315E-04	0.21211865830070E-04	0.50377632041769E-04
	1	-0.94505503911771E-05	-0.10045837732452E-03	0.68764980044275E-07
	2	0.10541122928199E-04	0.19096992239592E-04	-0.36660208861878E-07
	3	0.56040266518128E-04	0.86198259319230E-05	0.11686959861382E-06
	4	-0.17317904741240E-03	-0.37303028440467E-04	-0.52406357740558E-04
	5	0.16662991903909E-03	0.10482414957943E-03	0.23535269575505E-06
	6	0.11170213462198E-04	0.32391778709151E-05	-0.68268882634693E-07

Supplementary Table 1: Crystal field splitting parameters.

Crystal field splitting parameters(cm^{-1}) from CASSCF/RASSI/single_aniso in terms of Extended Stevens Operators after projection of the calculated levels onto the $|^7F_6>$, $|^6H_{15/2}>$ and $|^4I_{15/2}>$ ground multiplet for Tb^{III}, Dy^{III} and Er^{III}.

	E_1=0.0			E_6=563.8			E_{11}=753.8	
w.f.	***m_j***	**$\|c_i\|^2$**	***w.f.***	***m_j***	**$\|c_i\|^2$**	***w.f.***	***m_j***	**$\|c_i\|^2$**
1	±6	5.00×10^{-1}	6	±6	6.87×10^{-6}	11	±6	1.58×10^{-6}
	±5	1.60×10^{-7}		±5	2.77×10^{-5}		±5	1.29×10^{-6}
	±4	7.13×10^{-6}		±4	4.99×10^{-1}		±4	7.84×10^{-4}
	±3	3.63×10^{-6}		±3	3.33×10^{-4}		±3	2.96×10^{-3}
	±2	3.48×10^{-6}		±2	9.23×10^{-4}		±2	2.72×10^{-1}
	±1	1.02×10^{-6}		±1	2.72×10^{-5}		±1	5.13×10^{-2}
	0	1.47×10^{-7}		0	4.05×10^{-7}		0	3.46×10^{-1}
	$E_2=2.74\times10^{-4}$			E_7=680.3			E_{12}=771.6	
2	±6	5.00×10^{-1}	7	±6	4.59×10^{-6}	12	±6	1.26×10^{-6}
	±5	1.60×10^{-7}		±5	1.50×10^{-4}		±5	1.36×10^{-5}
	±4	7.14×10^{-6}		±4	2.75×10^{-4}		±4	1.04×10^{-3}
	±3	3.53×10^{-6}		±3	4.26×10^{-1}		±3	1.52×10^{-2}
	±2	3.47×10^{-6}		±2	3.23×10^{-3}		±2	1.45×10^{-1}
	±1	9.12×10^{-7}		±1	6.89×10^{-2}		±1	1.83×10^{-1}
	0	4.14×10^{-13}		0	2.52×10^{-3}		0	3.11×10^{-1}
	E_3=334.0			E_8=689.8			E_{13}=772.6	
3	±6	1.44×10^{-7}	8	±6	3.29×10^{-6}	13	±6	1.94×10^{-6}
	±5	5.00×10^{-1}		±5	1.01×10^{-4}		±5	2.35×10^{-5}
	±4	3.04×10^{-5}		±4	2.63×10^{-4}		±4	6.97×10^{-4}
	±3	1.09×10^{-4}		±3	4.94×10^{-1}		±3	1.21×10^{-2}
	±2	1.08×10^{-5}		±2	1.51×10^{-3}		±2	1.32×10^{-1}
	±1	2.51×10^{-5}		±1	4.24×10^{-3}		±1	2.88×10^{-1}
	0	2.43×10^{-5}		0	9.74×10^{-5}		0	1.34×10^{-1}
	E_4=334.0			E_9=718.7				
4	±6	1.41×10^{-7}	9	±6	3.90×10^{-6}			
	±5	5.00×10^{-1}		±5	9.57×10^{-6}			
	±4	2.98×10^{-5}		±4	3.12×10^{-3}			
	±3	1.12×10^{-4}		±3	8.38×10^{-4}			
	±2	8.47×10^{-6}		±2	4.32×10^{-1}			
	±1	2.82×10^{-5}		±1	1.01×10^{-2}			
	0	3.81×10^{-7}		0	1.07×10^{-1}			

	E_5=562.6			E_{10}=751.0	
5	±6	6.56×10^{-6}	10	±6	4.28×10^{-7}
	±5	2.97×10^{-5}		±5	1.11×10^{-5}
	±4	4.95×10^{-1}		±4	1.27×10^{-4}
	±3	3.69×10^{-4}		±3	4.80×10^{-2}
	±2	1.40×10^{-3}		±2	1.08×10^{-2}
	±1	1.54×10^{-4}		±1	3.95×10^{-1}
	0	6.22×10^{-3}		0	9.31×10^{-2}

Supplementary Table 2: Energy(cm^{-1}) levels and composition of wavefunctions for Tb as derived from CASSCF/RASSI/single_aniso calculations.

	E_1=0.0			E_5=121.6			E_9=363.3			E_{13}=544.4	
w.f.	***m_i***	**$\|c_i\|^2$**	***w.f.***	***m_i***	**$\|c_i\|^2$**	***w.f.***	***m_i***	**$\|c_i\|^2$**	***w.f.***	***m_i***	**$\|c_i\|^2$**
1	-7.5	8.56×10^{-2}	5	-7.5	0.00	9	-7.5	3.73×10^{-4}	13	-7.5	8.66×10^{-6}
	-6.5	8.80×10^{-1}		-6.5	6.23×10^{-10}		-6.5	8.78×10^{-5}		-6.5	5.89×10^{-5}
	-5.5	3.31×10^{-2}		-5.5	4.64×10^{-9}		-5.5	2.32×10^{-3}		-5.5	4.60×10^{-5}
	-4.5	1.22×10^{-3}		-4.5	2.82×10^{-9}		-4.5	1.12×10^{-1}		-4.5	8.12×10^{-5}
	-3.5	1.37×10^{-4}		-3.5	1.53×10^{-8}		-3.5	7.53×10^{-1}		-3.5	4.89×10^{-3}
	-2.5	4.56×10^{-6}		-2.5	3.29×10^{-8}		-2.5	1.17×10^{-1}		-2.5	1.35×10^{-1}
	-1.5	1.74×10^{-4}		-1.5	8.13×10^{-8}		-1.5	1.13×10^{-2}		-1.5	5.09×10^{-1}
	-0.5	7.21×10^{-6}		-0.5	1.03×10^{-7}		-0.5	7.07×10^{-4}		-0.5	1.54×10^{-1}
	0.5	4.86×10^{-7}		0.5	1.20×10^{-6}		0.5	2.54×10^{-3}		0.5	5.26×10^{-2}
	1.5	3.12×10^{-8}		1.5	4.34×10^{-5}		1.5	2.57×10^{-5}		1.5	1.42×10^{-1}
	2.5	6.36×10^{-8}		2.5	3.17×10^{-4}		2.5	9.95×10^{-5}		2.5	2.28×10^{-4}
	3.5	3.05×10^{-8}		3.5	4.61×10^{-4}		3.5	3.03×10^{-4}		3.5	1.93×10^{-3}
	4.5	4.83×10^{-9}		4.5	1.39×10^{-3}		4.5	1.60×10^{-5}		4.5	3.12×10^{-4}
	5.5	4.00×10^{-9}		5.5	1.00×10^{-2}		5.5	3.50×10^{-6}		5.5	2.88×10^{-5}
	6.5	2.13×10^{-11}		6.5	8.52×10^{-2}		6.5	7.09×10^{-9}		6.5	2.91×10^{-5}
	7.5	0.00		7.5	9.03×10^{-1}		7.5	0.00		7.5	0.00
	E_2=0.0			E_6=121.6			E_{10}=363.3			E_{14}=544.4	
2	-7.5	0.00	6	-7.5	9.03×10^{-1}	10	-7.5	0.00	14	-7.5	0.00
	-6.5	2.13×10^{-11}		-6.5	8.52×10^{-2}		-6.5	7.09×10^{-9}		-6.5	2.91×10^{-5}
	-5.5	4.00×10^{-9}		-5.5	1.00×10^{-2}		-5.5	3.50×10^{-6}		-5.5	2.88×10^{-5}
	-4.5	4.83×10^{-9}		-4.5	1.39×10^{-3}		-4.5	1.60×10^{-5}		-4.5	3.12×10^{-4}
	-3.5	3.05×10^{-8}		-3.5	4.61×10^{-4}		-3.5	3.03×10^{-4}		-3.5	1.93×10^{-3}
	-2.5	6.36×10^{-8}		-2.5	3.17×10^{-4}		-2.5	9.95×10^{-5}		-2.5	2.28×10^{-4}
	-1.5	3.12×10^{-8}		-1.5	4.34×10^{-5}		-1.5	2.57×10^{-5}		-1.5	1.42×10^{-1}
	-0.5	4.86×10^{-7}		-0.5	1.20×10^{-6}		-0.5	2.54×10^{-3}		-0.5	5.26×10^{-2}
	0.5	7.21×10^{-6}		0.5	1.03×10^{-7}		0.5	7.07×10^{-4}		0.5	1.54×10^{-1}
	1.5	1.74×10^{-4}		1.5	8.13×10^{-8}		1.5	1.13×10^{-2}		1.5	5.09×10^{-1}
	2.5	4.56×10^{-6}		2.5	3.29×10^{-8}		2.5	1.17×10^{-1}		2.5	1.35×10^{-1}
	3.5	1.37×10^{-4}		3.5	1.53×10^{-8}		3.5	7.53×10^{-1}		3.5	4.89×10^{-3}
	4.5	1.22×10^{-3}		4.5	2.82×10^{-9}		4.5	1.12×10^{-1}		4.5	8.12×10^{-5}
	5.5	3.31×10^{-2}		5.5	4.64×10^{-9}		5.5	2.32×10^{-3}		5.5	4.60×10^{-5}
	6.5	8.80×10^{-1}		6.5	6.23×10^{-10}		6.5	8.78×10^{-5}		6.5	5.89×10^{-5}
	7.5	8.56×10^{-2}		7.5	0.00		7.5	3.73×10^{-4}		7.5	8.66×10^{-6}
	E_3=78.0			E_7=221.5			E_{11}=472.6			E_{15}=590.5	
3	-7.5	0.00	7	-7.5	3.19×10^{-4}	11	-7.5	7.74×10^{-5}	15	-7.5	1.84×10^{-6}
	-6.5	3.61×10^{-8}		-6.5	1.18×10^{-3}		-6.5	1.29×10^{-4}		-6.5	1.47×10^{-5}
	-5.5	6.90×10^{-7}		-5.5	8.77×10^{-2}		-5.5	5.72×10^{-5}		-5.5	2.62×10^{-4}
	-4.5	1.60×10^{-7}		-4.5	7.96×10^{-1}		-4.5	3.00×10^{-3}		-4.5	3.56×10^{-4}
	-3.5	4.91×10^{-8}		-3.5	1.07×10^{-1}		-3.5	1.23×10^{-1}		-3.5	1.59×10^{-3}
	-2.5	1.05×10^{-8}		-2.5	6.60×10^{-3}		-2.5	7.14×10^{-1}		-2.5	2.34×10^{-2}
	-1.5	4.28×10^{-7}		-1.5	4.83×10^{-4}		-1.5	1.04×10^{-1}		-1.5	8.04×10^{-2}
	-0.5	9.24×10^{-6}		-0.5	4.61×10^{-4}		-0.5	2.55×10^{-2}		-0.5	7.50×10^{-1}
	0.5	1.75×10^{-4}		0.5	1.16×10^{-4}		0.5	5.68×10^{-5}		0.5	1.44×10^{-2}
	1.5	2.79×10^{-5}		1.5	1.44×10^{-6}		1.5	2.68×10^{-2}		1.5	1.25×10^{-1}
	2.5	2.62×10^{-4}		2.5	1.01×10^{-6}		2.5	3.09×10^{-4}		2.5	2.64×10^{-3}
	3.5	3.83×10^{-3}		3.5	1.09×10^{-6}		3.5	2.35×10^{-3}		3.5	1.57×10^{-3}
	4.5	8.46×10^{-2}		4.5	1.50×10^{-5}		4.5	1.95×10^{-4}		4.5	3.68×10^{-4}
	5.5	8.67×10^{-1}		5.5	3.75×10^{-6}		5.5	3.07×10^{-6}		5.5	7.03×10^{-7}
	6.5	3.34×10^{-2}		6.5	6.28×10^{-8}		6.5	8.13×10^{-6}		6.5	2.00×10^{-5}
	7.5	1.11×10^{-2}		7.5	0.00		7.5	0.00		7.5	0.00

	E_4=78.0			E_8=221.5			E_{12}=472.6			E_{16}=590.5	
4	-7.5	1.11×10^{-2}	8	-7.5	0.00	12	-7.5	0.00	16	-7.5	0.00
	-6.5	3.34×10^{-2}		-6.5	6.28×10^{-8}		-6.5	8.13×10^{-6}		-6.5	2.00×10^{-5}
	-5.5	8.67×10^{-1}		-5.5	3.75×10^{-6}		-5.5	3.07×10^{-6}		-5.5	7.03×10^{-7}
	-4.5	8.46×10^{-2}		-4.5	1.50×10^{-5}		-4.5	1.95×10^{-4}		-4.5	3.68×10^{-4}
	-3.5	3.83×10^{-3}		-3.5	1.09×10^{-6}		-3.5	2.35×10^{-3}		-3.5	1.57×10^{-3}
	-2.5	2.62×10^{-4}		-2.5	1.01×10^{-6}		-2.5	3.09×10^{-4}		-2.5	2.64×10^{-3}
	-1.5	2.79×10^{-5}		-1.5	1.44×10^{-6}		-1.5	2.68×10^{-2}		-1.5	1.25×10^{-1}
	-0.5	1.75×10^{-4}		-0.5	1.16×10^{-4}		-0.5	5.68×10^{-5}		-0.5	1.44×10^{-2}
	0.5	9.24×10^{-6}		0.5	4.61×10^{-4}		0.5	2.55×10^{-2}		0.5	7.50×10^{-1}
	1.5	4.28×10^{-7}		1.5	4.83×10^{-4}		1.5	1.04×10^{-1}		1.5	8.04×10^{-2}
	2.5	1.05×10^{-8}		2.5	6.60×10^{-3}		2.5	7.14×10^{-1}		2.5	2.34×10^{-2}
	3.5	4.91×10^{-8}		3.5	1.07×10^{-1}		3.5	1.23×10^{-1}		3.5	1.59×10^{-3}
	4.5	1.60×10^{-7}		4.5	7.96×10^{-1}		4.5	3.00×10^{-3}		4.5	3.56×10^{-4}
	5.5	6.90×10^{-7}		5.5	8.77×10^{-2}		5.5	5.72×10^{-5}		5.5	2.62×10^{-4}
	6.5	3.61×10^{-8}		6.5	1.18×10^{-3}		6.5	1.29×10^{-4}		6.5	1.47×10^{-5}
	7.5	0.00		7.5	3.19×10^{-4}		7.5	7.74×10^{-5}		7.5	1.84×10^{-6}

Supplementary Table 3: Energy(cm^{-1}) levels and composition of wavefunctions for Dy as derived from CASSCF/RASSI/single_aniso calculations.

	E_1=0.0			E_5=153.3			E_9=256.4			E_{13}=311.0	
w.f.	m_j	$\|c_i\|^2$	*w.f.*	m_j	$\|c_i\|^2$	*w.f.*	m_j	$\|c_i\|^2$	*w.f.*	m_j	$\|c_i\|^2$
1	-7.5	1.26×10^{-6}	5	-7.5	7.02×10^{-8}	9	-7.5	5.07×10^{-7}	13	-7.5	3.93×10^{-5}
	-6.5	3.26×10^{-11}		-6.5	1.36×10^{-1}		-6.5	8.47×10^{-1}		-6.5	1.19×10^{-10}
	-5.5	4.63×10^{-10}		-5.5	4.04×10^{-6}		-5.5	6.78×10^{-6}		-5.5	1.37×10^{-9}
	-4.5	3.98×10^{-7}		-4.5	2.47×10^{-9}		-4.5	9.79×10^{-10}		-4.5	2.80×10^{-1}
	-3.5	1.35×10^{-4}		-3.5	3.23×10^{-8}		-3.5	9.01×10^{-7}		-3.5	3.13×10^{-6}
	-2.5	1.06×10^{-9}		-2.5	8.42×10^{-1}		-2.5	1.34×10^{-1}		-2.5	5.87×10^{-10}
	-1.5	4.53×10^{-8}		-1.5	9.39×10^{-5}		-1.5	6.49×10^{-6}		-1.5	2.08×10^{-9}
	-0.5	1.13×10^{-4}		-0.5	3.09×10^{-9}		-0.5	7.48×10^{-10}		-0.5	4.70×10^{-4}
	0.5	9.98×10^{-1}		0.5	1.64×10^{-9}		0.5	1.71×10^{-10}		0.5	1.21×10^{-3}
	1.5	1.70×10^{-8}		1.5	1.51×10^{-2}		1.5	4.71×10^{-4}		1.5	1.11×10^{-9}
	2.5	3.45×10^{-9}		2.5	5.23×10^{-3}		2.5	2.38×10^{-3}		2.5	1.94×10^{-9}
	3.5	2.19×10^{-8}		3.5	1.36×10^{-8}		3.5	5.11×10^{-8}		3.5	1.86×10^{-6}
	4.5	1.68×10^{-3}		4.5	9.72×10^{-12}		4.5	4.45×10^{-11}		4.5	7.18×10^{-1}
	5.5	5.62×10^{-8}		5.5	6.35×10^{-4}		5.5	3.15×10^{-4}		5.5	2.03×10^{-8}
	6.5	4.08×10^{-10}		6.5	8.50×10^{-4}		6.5	1.50×10^{-2}		6.5	2.91×10^{-10}
	7.5	1.13×10^{-10}		7.5	1.36×10^{-13}		7.5	1.55×10^{-8}		7.5	1.33×10^{-5}
	E_2=0.0			E_6=153.3			E_{10}=256.4			E_{14}=311.0	
2	-7.5	1.13×10^{-10}	6	-7.5	1.36×10^{-13}	10	-7.5	1.55×10^{-8}	14	-7.5	1.33×10^{-5}
	-6.5	4.80×10^{-10}		-6.5	8.50×10^{-4}		-6.5	1.50×10^{-2}		-6.5	2.91×10^{-10}
	-5.5	5.62×10^{-8}		-5.5	6.35×10^{-4}		-5.5	3.15×10^{-4}		-5.5	2.03×10^{-8}
	-4.5	1.68×10^{-3}		-4.5	9.72×10^{-12}		-4.5	4.45×10^{-11}		-4.5	7.18×10^{-1}
	-3.5	2.19×10^{-8}		-3.5	1.36×10^{-8}		-3.5	5.11×10^{-8}		-3.5	1.86×10^{-6}
	-2.5	3.45×10^{-9}		-2.5	5.23×10^{-3}		-2.5	2.38×10^{-3}		-2.5	1.94×10^{-9}
	-1.5	1.70×10^{-8}		-1.5	1.51×10^{-2}		-1.5	4.71×10^{-4}		-1.5	1.11×10^{-9}
	-0.5	9.98×10^{-1}		-0.5	1.64×10^{-9}		-0.5	1.71×10^{-10}		-0.5	1.21×10^{-3}
	0.5	1.13×10^{-4}		0.5	3.09×10^{-9}		0.5	7.48×10^{-10}		0.5	4.70×10^{-4}
	1.5	4.53×10^{-8}		1.5	9.39×10^{-5}		1.5	6.49×10^{-6}		1.5	2.08×10^{-9}
	2.5	1.06×10^{-9}		2.5	8.42×10^{-1}		2.5	1.34×10^{-1}		2.5	5.87×10^{-10}
	3.5	1.35×10^{-4}		3.5	3.23×10^{-8}		3.5	9.01×10^{-7}		3.5	3.13×10^{-6}
	4.5	3.98×10^{-7}		4.5	2.47×10^{-9}		4.5	9.79×10^{-10}		4.5	2.80×10^{-1}
	5.5	4.63×10^{-10}		5.5	4.04×10^{-6}		5.5	6.78×10^{-6}		5.5	1.37×10^{-9}
	6.5	3.26×10^{-11}		6.5	1.36×10^{-1}		6.5	8.47×10^{-1}		6.5	1.19×10^{-10}
	7.5	1.26×10^{-6}		7.5	7.02×10^{-8}		7.5	5.07×10^{-7}		7.5	3.93×10^{-5}

	E_3=59.2			E_7=247.3			E_{11}=294.1			E_{15}=319.2	
3	-7.5	6.29×10^{-11}	7	-7.5	2.13×10^{-1}	11	-7.5	9.38×10^{-8}	15	-7.5	3.98×10^{-9}
	-6.5	1.47×10^{-6}		-6.5	1.05×10^{-6}		-6.5	6.72×10^{-7}		-6.5	2.50×10^{-10}
	-5.5	1.43×10^{-2}		-5.5	8.70×10^{-9}		-5.5	9.73×10^{-1}		-5.5	5.05×10^{-9}
	-4.5	2.56×10^{-10}		-4.5	1.68×10^{-7}		-4.5	1.31×10^{-8}		-4.5	4.64×10^{-5}
	-3.5	5.97×10^{-10}		-3.5	7.87×10^{-1}		-3.5	9.96×10^{-9}		-3.5	1.29×10^{-9}
	-2.5	1.99×10^{-5}		-2.5	2.32×10^{-7}		-2.5	2.22×10^{-6}		-2.5	1.18×10^{-9}
	-1.5	9.68×10^{-1}		-1.5	2.99×10^{-10}		-1.5	1.48×10^{-2}		-1.5	3.68×10^{-9}
	-0.5	9.84×10^{-9}		-0.5	5.84×10^{-11}		-0.5	6.05×10^{-8}		-0.5	2.15×10^{-5}
	0.5	4.34×10^{-8}		0.5	1.15×10^{-4}		0.5	2.85×10^{-9}		0.5	6.58×10^{-9}
	1.5	1.30×10^{-3}		1.5	1.54×10^{-8}		1.5	1.77×10^{-4}		1.5	1.89×10^{-9}
	2.5	1.57×10^{-2}		2.5	3.46×10^{-9}		2.5	2.23×10^{-4}		2.5	5.55×10^{-8}
	3.5	3.68×10^{-8}		3.5	1.98×10^{-7}		3.5	3.31×10^{-9}		3.5	2.13×10^{-1}
	4.5	1.85×10^{-9}		4.5	4.68×10^{-6}		4.5	1.12×10^{-8}		4.5	5.52×10^{-6}
	5.5	1.92×10^{-5}		5.5	9.74×10^{-9}		5.5	1.17×10^{-2}		5.5	8.94×10^{-8}
	6.5	5.99×10^{-4}		6.5	1.74×10^{-7}		6.5	1.06×10^{-4}		6.5	8.92×10^{-8}
	7.5	5.39×10^{-15}		7.5	5.41×10^{-8}		7.5	6.89×10^{-9}		7.5	7.87×10^{-1}
	E_4=59.2			E_8=247.3			E_{12}=294.1			E_{16}=319.2	
4	-7.5	5.39×10^{-15}	8	-7.5	5.41×10^{-8}	12	-7.5	6.89×10^{-9}	16	-7.5	7.87×10^{-1}
	-6.5	5.99×10^{-4}		-6.5	1.74×10^{-7}		-6.5	1.06×10^{-4}		-6.5	8.92×10^{-8}
	-5.5	1.92×10^{-5}		-5.5	9.74×10^{-9}		-5.5	1.17×10^{-2}		-5.5	8.94×10^{-8}
	-4.5	1.85×10^{-9}		-4.5	4.68×10^{-6}		-4.5	1.12×10^{-8}		-4.5	5.52×10^{-6}
	-3.5	3.68×10^{-8}		-3.5	1.98×10^{-7}		-3.5	3.31×10^{-9}		-3.5	2.13×10^{-1}
	-2.5	1.57×10^{-2}		-2.5	3.46×10^{-9}		-2.5	2.23×10^{-4}		-2.5	5.55×10^{-8}
	-1.5	1.30×10^{-3}		-1.5	1.54×10^{-8}		-1.5	1.77×10^{-4}		-1.5	1.89×10^{-9}
	-0.5	4.34×10^{-8}		-0.5	1.15×10^{-4}		-0.5	2.85×10^{-9}		-0.5	6.58×10^{-9}
	0.5	9.84×10^{-9}		0.5	5.84×10^{-11}		0.5	6.05×10^{-8}		0.5	2.15×10^{-5}
	1.5	9.68×10^{-1}		1.5	2.99×10^{-10}		1.5	1.48×10^{-2}		1.5	3.68×10^{-9}
	2.5	1.99×10^{-5}		2.5	2.32×10^{-7}		2.5	2.22×10^{-6}		2.5	1.18×10^{-9}
	3.5	5.97×10^{-10}		3.5	7.87×10^{-1}		3.5	9.96×10^{-9}		3.5	1.29×10^{-9}
	4.5	2.56×10^{-10}		4.5	1.68×10^{-7}		4.5	1.31×10^{-8}		4.5	4.64×10^{-5}
	5.5	1.43×10^{-2}		5.5	8.70×10^{-9}		5.5	9.73×10^{-1}		5.5	5.05×10^{-9}
	6.5	1.47×10^{-6}		6.5	1.05×10^{-6}		6.5	6.72×10^{-7}		6.5	2.5×10^{-10}
	7.5	6.29×10^{-11}		7.5	2.13×10^{-1}		7.5	9.38×10^{-8}		7.5	3.98×10^{-9}

Supplementary Table 4: Energy(cm^{-1}) levels and composition of wavefunctions for Er as derived from CASSCF/RASSI/single_aniso calculations.

References

[1] Stepanow, S. *et al.* Spin and Orbital Magnetic Moment Anisotropies of Monodispersed Bis(Phthalocyaninato)Terbium on a Copper Surface. *J. Am. Chem. Soc.* **2010,** *132,* 11900-11901.

[2] Margheriti, L. *et al.* X-Ray Detected Magnetic Hysteresis of Thermally Evaporated Terbium Double-Decker Oriented Films. *Adv Mater.* **2010,** *22,* 5488-5493.

[3] Biagi, R. *et al.* X-ray Absorption and Magnetic Circular Dichroism Investigation of Bis(phthalocyaninato) Terbium Single-Molecule Magnets Deposited on Graphite. *Phys. Rev.B* **2010,** *82,* 224406.

[4] Wende, H. *et al.* Substrate-Induced Magnetic Ordering and Switching of Iron Porphyrin Molecules. *Nat. Mater.* **2007,** *6,* 516-520.

[5] Bernien, M. *et al.* Tailoring the Nature of Magnetic Coupling of Fe-Porphyrin Molecules to Ferromagnetic Substrates. *Phys. Rev. Lett.* **2009,** *102,* 047202.

[6] Katoh, K. *et al.* Direct Observation of Lanthanide(III)-Phthalocyanine Molecules on Au(111) by Using Scanning Tunneling Microscopy and Scanning Tunneling Spectroscopy and Thin-Film Field-Effect Transistor Properties of Tb(III)- and Dy(III)-Phthalocyanine Molecules. *J. Am. Chem. Soc.* **2009,** *131,* 9967-9976.

[7] Komeda, T. *et al.* Observation and Electric Current Control of a Local Spin in a Single-Molecule Magnet. *Nat. Commun.* **2011,** *2,* 217.

[8] Lopes, M. *et al.* Surface-Enhanced Raman Signal for Terbium Single-Molecule Magnets Grafted on Graphene. *ACS Nano* **2010,** *4,* 7531-7537.

[9] Ling, X. *et al.* Can Graphene be Used as a Substrate for Raman Enhancement? *Nano Lett.* **2010,** *10,* 553-561.

[10] Chibotaru, L. F. *et al.* The Origin of Non-Magnetic Kramers Doublets in the Ground State of Dysprosium Triangles: Evidence for a Toroidal Magnetic Moment. *Angew. Chem. Int. Ed.*, **2008,** *47,* 4126-4129.

[11] Chibotaru, L. F. *et al.* Structure, Magnetism, and Theoretical Study of a Mixed Valence Co Heptanuclear Wheel: Lack of SMM Behavior Despite Negative Magnetic Anisotropy. *J. Am. Chem. Soc.*, **2008,** *130,* 12445-12455.

[12] Chibotaru, L. F. & Ungur, L. Ab Initio Calculation of Anisotropic Magnetic Properties of Complexes: Unique Definition of Pseudospin Hamiltonians and Their Derivation. *J. Chem. Phys.* **2012,** *137,* 064112.

[13] Ungur, L. *et al.* Interplay of Strongly Anisotropic Metal Ions in Magnetic Blocking of Complexes. *Inorg. Chem.* **2013,** *52,* 6328.

[14] Karlström, G. *et al.* MOLCAS: a Program Package for Computational Chemistry. *Computational Material Science* **2003,** *28,* 222-239.

[15] Marx, R. *et al.* Spectroscopic Determination of Crystal Field Splittings in Lanthanide Double Deckers. *Chem. Sci*. **2014,** *5,* 3287.

[16] Hess, B. A. *et al. Chem. Phys. Lett.* **1996,** *251,* 365.

[17] Schimmelpfennig, B. *AMFI, an Atomic Mean-Field Spin-Orbit Integral Program*. Stockholm University (1996).
